# Editorial: Consequences of Iodine Deficiency in Pregnancy

**DOI:** 10.3389/fendo.2021.740239

**Published:** 2021-07-28

**Authors:** Sun Y. Lee

**Affiliations:** Section of Endocrinology, Diabetes, and Nutrition, Boston University School of Medicine, Boston, MA, United States

**Keywords:** iodine, pregnancy, iodine deficiency, iodine deficiency in pregnancy, hypothyroidism in pregnancy

## Introduction

Iodine is an essential micronutrient for thyroid hormone synthesis. Adequate thyroid hormone is critical for normal fetal development. As the fetal thyroid gland does not mature until mid-gestation, the fetus depends solely on maternal thyroid hormone crossing the placenta during early stages of pregnancy (1). Maternal overt hypothyroidism is associated with adverse pregnancy and child developmental outcomes (2, 3). However, the effects of maternal subclinical hypothyroidism have been debated. In this Research Topic, Chen et al. explored potential associations between maternal thyroid function and birth outcomes in 8,985 Chinese mother-child pairs. Serum thyroid stimulating hormone (TSH), free thyroxine (FT4), and thyroid peroxidase (TPO) antibody levels were measured. Rates of cesarean section, preterm birth, neonatal hyperbilirubinemia, and low birth weight (LBW) were assessed. Children born to mothers with TSH > 4.0mIU/L had a two-fold higher risk of LBW compared to those born to mothers with TSH 0.1-2.5mIU/L, regardless of TPO antibody status. This finding is in line with previous studies showing adverse effects of maternal subclinical hypothyroidism on fetal growth (4). However, there were no significant associations between maternal subclinical hypothyroidism and risks of other birth outcomes, in contrast to the increased risk of preterm birth seen in several previous studies (5, 6). Conflicting reports regarding the effects of maternal subclinical hypothyroidism on pregnancy outcomes may be due to variability in timing of thyroid function measurements, iodine status of the study populations, and assessment of TPO antibody status among studies.

Given the importance of adequate thyroid hormone, adequate maternal iodine nutrition is essential during pregnancy. There is an increased demand for iodine in pregnancy due to increased thyroid hormone production, increased urinary iodine losses, and transplacental transport of iodine for fetal thyroid hormone synthesis (7). Consequently, the World Health Organization (WHO) recommends a higher iodine intake of 250µg/day in pregnant women, compared to 150µg/day in non-pregnant adults (8). Despite global efforts to implement salt iodization by international organizations such as the Iodine Global Network (IGN), UNICEF, and WHO, iodine deficiency remains a leading cause of maternal hypothyroidism worldwide. Recent data from IGN showed that pregnant women had inadequate iodine status in 23 out of 34 countries assessed (9), and 35 million newborns are estimated to be unprotected from adverse consequences of iodine deficiency worldwide. In this Research Topic, Toloza et al. reviewed consequences of severe iodine deficiency in pregnancy. Maternal severe iodine deficiency is associated with adverse pregnancy outcomes such as miscarriage, stillbirth, neonatal mortality, and growth retardation. It also affects neonatal and offspring development since thyroid hormone is essential for normal brain development (10, 11). As discussed in the review by Toloza et al., the effects of maternal severe iodine deficiency on offspring neurocognitive development are most dramatically demonstrated by cretinism and its eradication with iodine supplementation (12, 13).

However, the effects of mild-to-moderate iodine deficiency in pregnancy are more variable. Some studies have shown increased risks of miscarriage (14), preterm labor (15–17), and LBW (15, 16), and impaired language (18) and fine motor development (19) in children of mild-to-moderately iodine deficient mothers. In contrast, other studies reported no significant association between maternal iodine status and adverse pregnancy outcomes (20) or verbal skills in children (21). In this Research Topic, Schiller et al. assessed iodine status and thyroid function of 100 healthy Israeli pregnant women in their first trimester. Women in this study had moderate iodine deficiency with a median urinary iodine concentration (UIC) of 49µg/L. Iodine-containing supplement use was strongly correlated with UIC, although 53% of women taking iodine-containing supplements still had UIC < 100µg/L. However, there were no significant associations between maternal UIC in the first trimester and maternal or neonatal thyroid hormone levels. There were no significant associations between maternal UIC levels and gestational age at birth or birth weight, similar to a previous U.K. study (20).

There are only a few studies assessing the impact of maternal iodine supplementation on pregnancy or childhood developmental outcomes. Some studies showed higher developmental scores in children of women with iodine supplementation, while others did not (22, 23). In this issue, Verhagen et al. assessed the effects of iodine supplementation on maternal thyroid function and child development in a mildly iodine deficient population. A total of 514 Thai pregnant women were randomized to either 200µg/day of iodine or placebo from the first trimester to delivery. The median UIC at recruitment was 112µg/L, indicating mild iodine deficiency. Iodine-treated mothers had a slightly greater decrease in FT4 levels compared to placebo-treated mothers, although all remained within normal range. At 5.7 years of age, there were no significant differences in developmental assessment scores between children in the iodine group and children in the placebo group. It is notable that mothers in placebo group also achieved iodine sufficiency in the second and third trimesters, which may have contributed to the lack of treatment effect. Evidence regarding the benefit of maternal iodine supplementation in pregnancy is currently limited by the variable exposure time during pregnancy, with initiation of iodine supplementation later in pregnancy in some cases, and lack of significant differences in iodine status of pregnant women between iodine supplemented and control groups in some available studies.

This Research Topic explored topics related to iodine nutrition in pregnancy. Adequate thyroid hormone and iodine nutrition are important in pregnancy, as evidenced by adverse effects of severe iodine deficiency on pregnancy and child neurodevelopmental outcomes. However, the effects of mild-to-moderate iodine deficiency in pregnancy are less clear. A few intervention trials on potential benefits of maternal iodine supplementation in pregnancy have shown a lack of significant benefit, but these trials have had several methodologic limitations. Given the currently available evidence, many societies including the American Thyroid Association, the European Thyroid association, and the Endocrine Society recommend supplementation with 150µg/day of iodine in women who are planning to become pregnant or are pregnant. Still, larger cohort studies and interventional trials are needed to further elucidate the effects of mild-to-moderate maternal iodine deficiency in pregnancy and the potential impact of iodine supplementation.

## Author Contributions

The author confirms being the sole contributor of this work and has approved it for publication.

## Funding

SYL is supported in part by NIH 1K23ES028736-01A1 and Boston University School of Medicine Department of Medicine Career Investment Award.

## Conflict of Interest

The author declares that the research was conducted in the absence of any commercial or financial relationships that could be construed as a potential conflict of interest.

## Publisher’s Note

All claims expressed in this article are solely those of the authors and do not necessarily represent those of their affiliated organizations, or those of the publisher, the editors and the reviewers. Any product that may be evaluated in this article, or claim that may be made by its manufacturer, is not guaranteed or endorsed by the publisher.
